# Lemann Index for Assessment of Crohn’s Disease: Correlation with the Quality of Life, Endoscopic Disease Activity, Magnetic Resonance Index of Activity and C- Reactive Protein

**DOI:** 10.1515/med-2019-0092

**Published:** 2019-11-07

**Authors:** Vestina Straksyte, Gediminas Kiudelis, Irina Gineikiene, Dainius Janciauskas, Algidas Basevicius, Saulius Lukosevicius, Limas Kupcinskas

**Affiliations:** 1 Department of Radiology, Lithuanian University of Health Sciences, Eivenių str. 2, Kaunas, Lithuania; 2 Department of Gastroenterology, Lithuanian University of Health Sciences, Kaunas, Lithuania; 3 Department of Pathological Anatomy, Lithuanian University of Health Sciences, Kaunas, Lithuania

**Keywords:** Crohn’s disease, Lemann index, Magnetic resonance enterocolonography, Quality of life

## Abstract

**Aim:**

Crohn’s disease (CD) is characterized by continuing inflammation and progressive gut damage. Despite many scoring indices of CD, there is a lack of more global assessment tools for the evaluation of the total disease impact on the gut.

**Methods:**

Fift y-three adult patients with proven CD underwent magnetic resonance enterocolonography (MR-EC), colonoscopy, and clinical activity assessment, including CRP. Quality of life was assessed using IBDQ. MR-EC was used to evaluate the Magnetic Resonance Index of Activity (MaRIA- global (G)) and the Lemann Index (LI). The CD Endoscopic Index of Severity (CDEIS) was used to score the endoscopic activity of the disease.

**Results:**

A signifi cant correlation between the LI and IBDQ was found (r=-0.812, P<0.01). LI and MaRIA-G correlated moderately, while the LI did not correlate significantly with CRP and CDEIS. For the detection of endoscopically active CD, MaRIA-G was more sensitive and specific (83.3%; 73.3%) compared to the LI (66.7%; 60.0%). There was a moderate correlation between CRP and MaRIA-G, as well as CRP and CDEIS (r=0.496; r=0.527,<0.01).

**Conclusion:**

A signifi cant negative correlation between the LI and quality of life, measured by IBDQ, was found in our study, suggesting that the LI could resemble more global features of the disease, besides inflammatory activity of the gut.

## 1 Introduction

Crohn’s disease (CD) is an idiopathic, chronic inflammatory bowel disease with an increasing incidence [1]. A distinguishable feature of CD is a transmural intestinal inflammation of the gastrointestinal tract anywhere from the mouth to the anus [2]. CD usually presents early in life and can disturb social life, learning, career, and family planning [3]. Diagnostic delay is common in CD, and the inflammation frequently presents many years before the actual diagnosis is made [4].

A thorough evaluation of the small and large bowel with an optimal diagnostic tool such as magnetic resonance enterocolonography (MR-EC) may lead to the earlier detection of CD phenotype in the most of the patients, especially when the small bowel disease predominates [5]. Also, MR-EC is significant in identifying and managing complications such as fistulas, strictures, and abscesses [2].

Grading the activity of CD is significant for the objective evaluation of the disease’s course/progress and extent, as well as for monitoring the effectiveness of treatment [6,7]. Several indices and scores are developed to describe the activity and severity of the disease and patient’s quality of life. The most well-known tools for assessing disease activity and progression, as well as the quality of life, are - the Inflammatory Bowel Disease Questionnaire (IBDQ) [8], the CD Endoscopic Index of Severity (CDEIS) [9] and the Magnetic Resonance Index of Activity (MaRIA) [10].

The Lemann index (LI), has been developed recently, aiming to assess total gut damage score in CD [11]. It connects clinical, surgical, endoscopic, and imaging findings from all digestive tract segments into one composite score [12].

Our study aimed to disclose how LI correlates with IBDQ, MaRIA- global (G), CDEIS, and a routine inflammatory marker C-Reactive Protein (CRP).

## 2 Methods

### 2.1 Study design

We performed a single-center cross-sectional study in the departments of Radiology and Gastroenterology between June 2015 and January 2017.

The study was approved by the local Bioethics Committee (Protocol No. BE-2-48). Informed written consent was obtained from all the patients.

Inclusion criteria were the following: only adult (>18 years) patients with clinically symptomatic CD, a complete ileocolonoscopy and MR-EC examinations, an MR-EC performed within 14 days from ileocolonoscopy.

Exclusion criteria were pacemakers, metal devices, prostheses or foreign bodies in the patient’s body, and claustrophobia.

**Figure 1:**
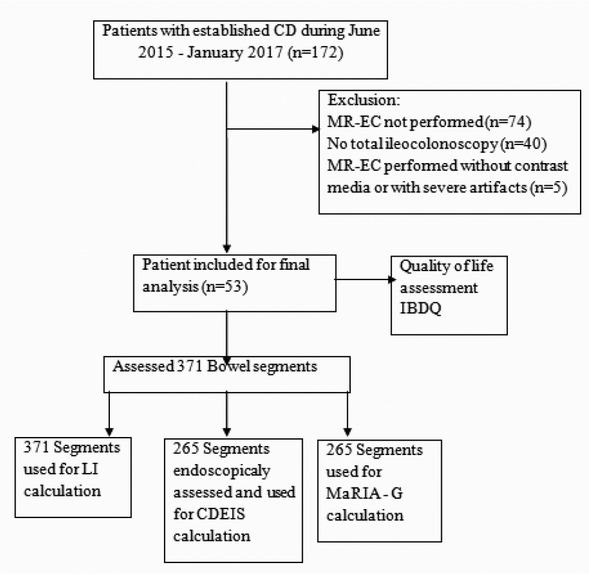
Flowchart of patient enrollment, exclusion criteria, and final study population. Abbreviations: CD– Crohn’s disease; CDEIS– Crohn's Disease Endoscopic Index of Severity; IBDQ– inflammatory bowel disease questionnaire; LI– Lemann Index; MaRIA-G– Magnetic Resonance Index of Activity Global; MR-EC –Magnetic Resonance Enterocolonography.

Out of 172 patients diagnosed with CD, 53 fulfilled the inclusion criteria as mentioned above and were enrolled for further analysis (Figure 1). All 53 patients underwent clinical assessment, CRP testing, and filled in an IBDQ. Endoscopic disease activity was assessed using CDEIS [9].

According to the Montreal classification, we assessed CD location and behavior [13].

### 2.2 The protocol of MRI enterocolonography

All MR-EC were performed by using a 1.5 Tesla MR unit (Siemens Medical Systems, Erlangen, Germany) using the manufacturer’s phased-array body coils in the prone position. The patients were asked to take a bowel cleaning agent at personalized doses to cleanse the bowel and to fast overnight before the examination.

On the examination day, about 60 minutes before performing the test, each patient received orally 2,5% -1500-2000 ml solution of mannitol. In order to prevent peristalsis, 20 mg/ml N-Butyl Scopolamine (Buscopan, Boehringer, Ingelheim, Germany) was injected intravenously just before starting MR-EC. The applied MR-EC protocol: coronal and axial T2- weighted, coronal true fast imaging with steady-state (True-FISP), unenhanced and contrast-enhanced coronal, and axial T1- weighted images. All the patients tolerated MR-EC well. No adverse reactions were observed.

### 2.3 Image interpretation

The bowel was divided into seven segments: jejunum, proximal ileum, terminal ileum, caecum/ascending colon, transverse colon, descending colon/sigmoid, and rectum.

To quantify the extent of inflammation of the small bowel and colon, each segment was evaluated for mural wall thickness in millimeters (≥ 3mm estimated as thickening), the presence of mural edema (hyperintensity on T2-weighted images relative to the psoas muscle signal [10]), and mural contrast enhancement at the moment of 70 seconds after contrast admission. Inflamed segment length in centimeters was also measured. Ulcers were defined as deep impressions in the mucosal surface of the thickened bowel wall. Stenosis was stated as luminal narrowing in the CD affected segment without or with pre-stenotic dilatation. Phlegmon and fistulae were also evaluated.

### 2.4 Assessment of Quality of life

The IBDQ is a validated disease-specific quality of life assessment instrument for adults [14]. This questionnaire includes four main categories. These domains evolve gut symptoms, and systemic complaints, emotional and social functions [14]. The IBDQ consists of 32 questions. The response for each item is graded on a 7-point Likert scale, ranging from 1 (reflects the “worst” condition) to 7 (reflects the “best” condition). The total IBDQ score range from 32 to 224, highest scores implying for the best quality of life [15].

### 2.5 Assessment of bowel damage

The LI is a new innovative index aiming to assess cumulative digestive tract damage using MR-EC as a diagnostic tool [16]. Calculation includes the esophagus, stomach, duodenum, small, and large bowel [17]. Each segment is graded for stricturing and penetrating lesions according to severity, and also includes the history of surgical resections [16]. When applying the LI analysis, the gastrointestinal tract was divided into segments: upper tract (esophagus, stomach, duodenum), small bowel – 20 segments, colon/ rectum – 6 segments, anus – 1 segment. The bowel segments were recalculated according to the LI calculation instructions. The LI was assessed based on the following three visible features: stricturing lesions, penetrating lesions, and the history of surgery. For each element, grading from 0 (none) to 3 was performed [17], and 10 for each resected segment was added. The LI can range from 0 – as “no bowel damage,” to 140, - as “the heaviest bowel damage” [12].

### 2.6 CD activity evaluation

MaRIA is the first developed MRI index for grading CD activity and severity [19]. When developing it, CDEIS was used as the reference standard [20].

MaRIA was calculated according to the formula by Rimola et al. [10]. MaRIA Global (MaRIA-G) was calculated as the sum of all the segments of each patient. MaRIA (segment) =1.5×wall thickness (mm)+0.02×RCE+5×edema+10×ulceration. The Relative contrast enhancement (RCE) was calculated according to the following formula: RCE=[(wall signal intensity (WSI) postgadolinium–WSI pre-gadolinium)/(WSI pre-gadolinium)]×100×(SD noise pre-gadolinium/SD noise postgadolinium) [10].

### 2.7 Analysis of Endoscopy

The endoscopy as the gold standard for the evaluation of lesions in the colon and terminal ileum was performed by an experienced gastroenterologist, who was blinded to the MR-EC results. The conventional colonoscopy and upper endoscopy (gastroduodenoscopy) were performed through standard equipment (model CFQ 140; Olympus, Tokyo, Japan). Suspicious inflammatory segments were recorded and biopsied. All tissue sections were stained with hematoxylin and eosin, according to a standard protocol of the hospital.

For the CDEIS calculation, the presence or absence of deep and superficial ulceration, and the percentage of surface affected by ulcerations were evaluated. Also, ulcerated stenosis and non-ulcerated stenosis was assessed. All the factors were summed; thus higher scores indicated a more severe disease (total score ranges from 0 to 44) [21].

### 2.8 Statistical analysis

The statistical analysis was performed using the SPSS software package for Windows V20.0 (Statistical package for the social sciences, Chicago, Illinois, USA).

The normality assumption of data was verified with the Shapiro-Wilk test. Activity indices scores were compared using non-parametric Spearman‘s correlation. Correlation coefficients were interpreted accordingly, r between 0.0-0.2 was considered as insignificant, 0.2-0.4 as a weak, 0.4-0.7 as a moderate, 0.7-0.9 as a strong, 0.9-1.0 as a very strong correlation [22].

Areas under the receiver operating characteristic (ROC) curve were calculated and points for the best specificity and sensitivity established.

Statistical significance was assumed at a P value of <0.05.

## 3 Results

Fifty-three patients fulfilled the inclusion criteria. The demographic and clinical characteristics of the patients

are presented in Table 1. More than half of the investigated population were male, the mean age of the patients was 37 ±14.4 years. Disease location was mostly ileal (n=23, 43.4%) and ileocolonic (n=23, 43.4%). Disease behavior prevalently was non-stricturing, non- penetrating (n=31, 58.5 %). None of the patients had a history of surgical resection and perianal disease.

**Table 1:** Demographic and clinical data of the patient population.

Characteristics	Crohn Disease (n= 53)
Male, n (%)	32 (60.4)
Female, n (%)	21 (39.6)
Age at inclusion mean (SD), years	37 ± 14.4
Disease duration at inclusion mean (SD), years	4.2 ± 2.3
Disease location	
L1- terminal ileum, n (%)	23 (43.4)
L2- colonic, n (%)	7 (13.2 )
L3- ileocolonic, n (%)	23 (43.4)
L4- isolated upper disease n (%)	0(0)
Disease behavior	
B1- non-stricturing, non- penetrating	31 (58.5)
B2- stricturing	11 (20.75)
B3- penetrating	11(20.75)
Previous surgery	
None, n (%)	53(100)
Tobacco use	
Never, n (%)	42 (79.24)
Previous, n (%)	7 (13.22)
Current, n (%)	4 (7.54)
C reactive protein mean (SD ), nmol/l	305.33 ± 76.66

**Figure 2:**
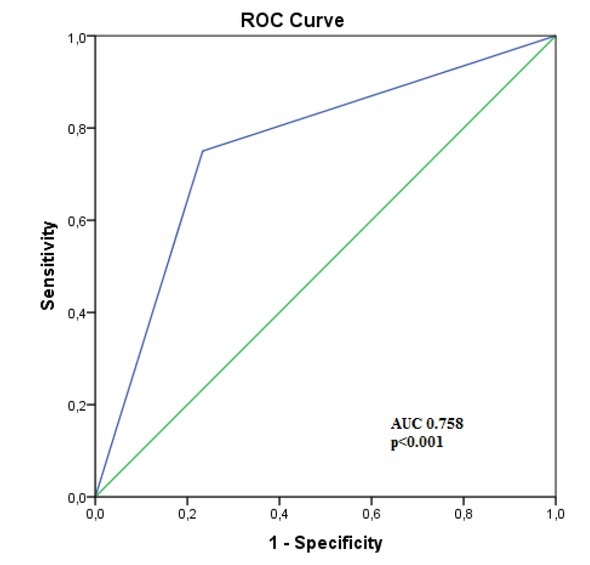
ROC curve analysis of MR-EC in predicting endoscopic Crohn’s disease activity.

The sensitivity and specificity of the MR-EC in detecting CD lesions using endoscopy as the gold standard were respectively: 74.58% and 77.32 % (Figure 2).

**Figure 3:**
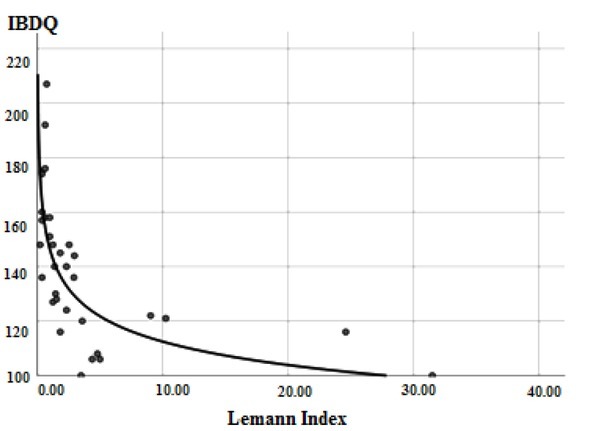
Correlation of Lemann Index and the Inflammatory Bowel Disease Questionnaire.

A strong negative correlation was found between the LI and the IBDQ (r= -0.812, P<0.01, Figure 3).

**Figure 4:**
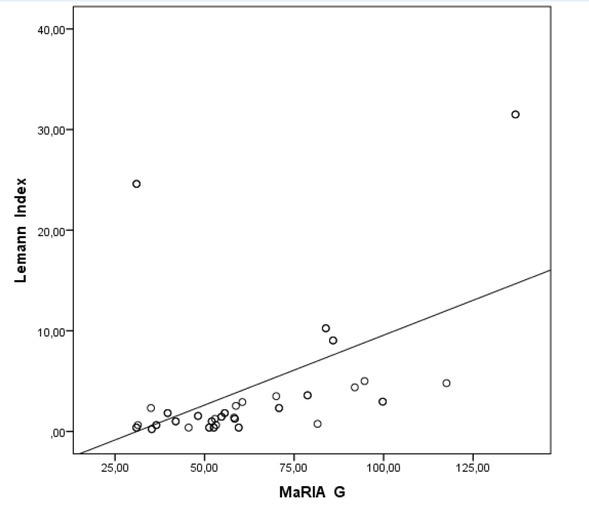
Correlation of Lemann Index and Magnetic Resonance Index of Activity Global.

Moreover, there was a moderate correlation between MaRIA-G and CDEIS, also MaRIA-G and the LI (Figure 4), respectively (r=0.685 and r=0.458, P<0.01). There was no significant correlation between the LI and CDEIS, as well as the IBDQ and CDEIS.

We also evaluated the value of MR-EC indices in detecting endoscopically active disease. CDEIS ≥9 was

considered as a cut off value for identifying the active disease.

**Figure 5:**
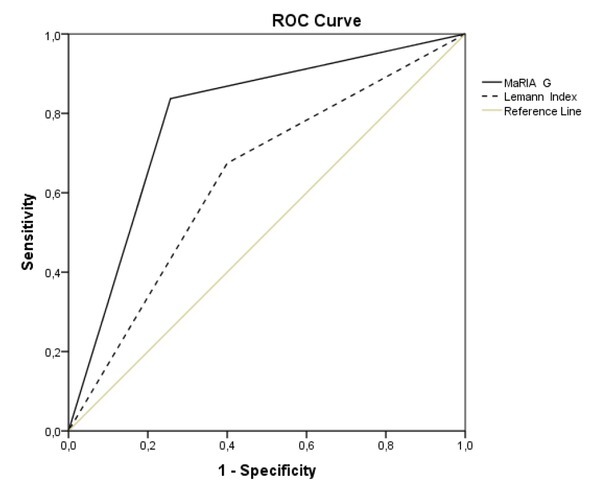
The sensitivity and specificity of Magnetic Resonance Index of Activity Global and the Lemann Index in detecting active Crohn’s Disease at endoscopy (Crohn’s Disease Endoscopic Index of Severity ≥ 9, P<0.01).

All the results of the indices mentioned above demonstrated acceptable values for detecting disease activity. Among the indices, MaRIA-G had higher sensitivity than the LI (83.3% vs. 66.7%, P<0.01) and specificity (73.3% vs. 60.0%, P<0.01). The accuracy was also higher for MaRIA-G compared to the LI (78.8% vs. 63.6%, P<0.01) (Figure 5).

CRP is the most widely used inflammatory marker for CD. We looked at the CRP correlation with other CD activity indices. There was a moderate correlation between

CRP and MaRIA–G (r=0.496, P<0.01) (Figure 6A). Also, there was a moderate correlation between CDEIS and CRP (r=0.527, P<0.01) (Figure 6B). However, there was no statistically significant correlation between CRP and LI, as well as with IBDQ.

**Figure 6A:**
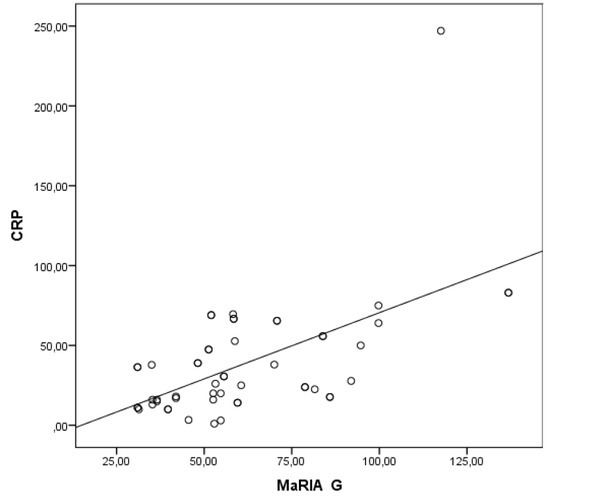
Correlation between CRP and Magnetic Resonance Index of Activity Global. Abbreviations: CDEIS– Crohn’s Disease Endoscopic Index of Severity; CRP- C-reactive protein.

**Figure 6B:**
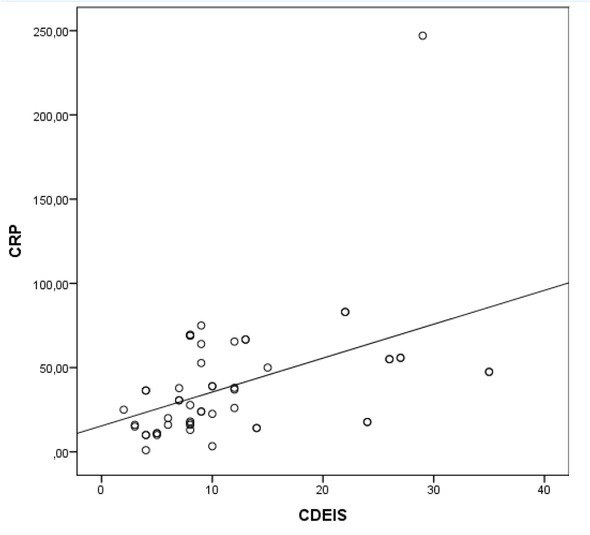
Correlation between CRP and Crohn’s Disease Endoscopic Index of Severity. Abbreviations: CDEIS– Crohn’s Disease Endoscopic Index of Severity; CRP- C-reactive protein.

## 4 Discussion

Our study demonstrated the MR-EC sensitivity of 74.58% and specificity of 77.32% in detecting CD lesions using endoscopy as the gold standard. Khaters et al. showed a slightly higher sensitivity of 82% and specificity 80% [23], Rieder et al. found MR-EC sensitivity and specificity 78% and 85% respectively [24]. Thus, our study demonstrated acceptable predictive values of MR-EC in detecting lesions of CD, comparable to findings by other authors.

To be noted, we did not find data about Crohn’s Disease Digestive Damage Score – the LI and patients’ quality of life measured by IBDQ. Our study found that increasing bowel damage evaluated by the LI was associated with decreasing IBDQ. This finding suggests that the LI could be used for a more global assessment of CD and could even assess the level of disability [12]. Knowles et al. observed that the quality of life assessment is significantly weaker for individuals when their disease is active compared to when it is quiescent. The quality of life could also be affected by the mental and emotional status of patients [25].

As there was no correlation between MaRIA-G and the IBDQ, we can assume that the quality of life does not always depend on inflammatory changes assessed by imaging methods per se. Stricturing and penetrating lesions that are more significant when calculating the LI could be more critical for IBDQ score. MaRIA index includes parameters resembling active inflammation of the gut [10], but such complications as strictures and fistulas are not scored.

Rozendor et al. have found that the LI, which includes stricturing and penetrating characteristics of the disease, had a better value for prognosing surgery than MaRIA [20].

CDEIS also did not correlate with the IBDQ and the LI. Jauregui-Amezaga et al. established that patients with endoscopically severe inflammation may still be asymptomatic [26]. The quality of life of CD patients is likely to depend on many different factors.

The CDEIS and MaRIA correlation in our study is lower (r= 0.685) than reported by Rimola et al. (r=0.8) [10], but similar to the one estimated by Coimbra et al. (r=0.63) [27], Kim et al. (r=0.737) [28] and Sato et al. (r=0.6) [29]. Kim et al. noticed that different phases with contrast media could pervert MaRIA calculating results [28].

The prognostic values of MaRIA-G for detecting endoscopic lesions were slightly higher than LI (Figure 5). The possible reason for this could be that MaRIA is based on similar characteristics like the CDEIS is. The LI evolves the whole gut from mouth to anus per segment and analyses not only inflammatory parameters but includes cross-sectional stricturing and penetrating lesions, which are often missed while calculating CDEIS.

Pita et al. investigated the significant drawbacks of the LI, which are the complexity and need of multiple examinations for complete structural evaluation (upper and lower endoscopy and cross-sectional abdominal and pelvic imaging) [16].

We did not detect a correlation between CRP and the LI as well as CRP and the IBDQ. However, CRP and the CDEIS correlation were moderate. This result assumes that CRP is a nonspecific and straightforward biomarker of inflammation and is beneficial in assessing and monitoring disease activity, but is not able to describe more global structural damage of the disease [30].

Our study had some strengths: a well designed clinical study, data were collected prospectively. Also, we were evaluating the LI as a new tool for CD activity assessment. In our opinion, it was the first study where the LI was correlated with other widely used CD activity indices. The limitation of the investigation might be the fact that according to the incidence, a relatively small group of patients were investigated; however, we presume that a large multicenter study would show more precise results.

In conclusion, MR-EC sensitivity and specificity for predicting endoscopically active CD lesions were higher when using MaRIA index compared to the LI. We found a strong negative correlation between the LI and quality of life measured by the IBDQ. This correlation was not observed when using MaRIA index. Therefore the LI could be more helpful in assessing more global characteristics of the disease. CRP showed good correlation with the CDEIS and MaRIA, but not with the LI and the IBDQ, confirming its role as a beneficial biomarker for assessing disease activity.
